# Posterior wall penetration of the internal jugular vein during central venous catheter insertion using real-time ultrasound: Two case reports

**DOI:** 10.1097/MD.0000000000022122

**Published:** 2020-09-11

**Authors:** Jeong Eun Lee, Myeong Jin Kim, Kyung-Hwa Kwak

**Affiliations:** Department of Anesthesiology and Pain Medicine, School of Medicine, Kyungpook National University, Kyungpook National University Hospital, Daegu, South Korea.

**Keywords:** central venous catheter, malposition, ultrasound

## Abstract

**Rationale:**

Because central venous catheters (CVCs) are placed at the great vessels, mechanical complications can be fatal. Using the landmark method alone can make CVC difficult to access, depending on the skill of the operator and various patient conditions, such as anatomical variations of the vessels, young age, hypovolemic state, obesity, and short neck. Therefore, ultrasound (US)-guided techniques, including visualization of the vein and needle in the lumen of the vessel, are recommended. Nevertheless, our experience demonstrated that CVC malposition or vascular penetration cannot be prevented completely, even with real-time US guidance.

**Patient concerns:**

The first patient was a 19-year-old woman (weight = 58 kg, height = 155 cm) who underwent CVC cannulation in the right internal jugular vein (IJV) under general anesthesia using real-time US. The second patient, a 50-year-old woman (weight = 51.6 kg, height = 155.7 cm), underwent CVC insertion in the right IJV using real-time US.

**Diagnoses:**

During guidewire insertion in the first case, the posterior wall of IJV was penetrated, and a break in the core body of the guidewire was detected. In the case of second patient, CVC was embedded in the posterior wall of IJV and misplaced in the interpleural space in the right thorax. In both cases, an out-of-plane US approach was used.

**Interventions:**

In the first case, the broken guidewire was completely removed with real-time US guidance. In the second case, all fluid injected through CVC was aspirated, and then CVC was removed.

**Outcomes:**

In both cases, surgeries were completed successfully and all the patients were discharged without any complications.

**Lessons:**

Even if the needle tip is located in the lumen of IJV and blood aspiration is confirmed on real-time US, vascular penetration or CVC malposition during the procedure cannot be completely prevented because of the limitation of the US imaging field. These results suggest that care must be exercised even during US-guided CVC placement and that alternative US-guided techniques or supplementary monitoring should be considered to confirm proper CVC position.

## 1 Introduction

Central venous catheters (CVCs) are often used for perioperative intensive care in the anesthesiology department. CVC insertion is an invasive procedure that can result in mechanical complications such as malposition, pneumothorax, arterial puncture, nerve injury, arrhythmia, great vessel rupture, and cardiac puncture. Mechanical complications during CVC insertion can occur during every step of the procedure and can be related to the operator's skill, needle, guidewire, dilator, or CVC itself. In patients with coagulation disorders or anatomical variations, CVC insertion using the anatomical landmark technique alone is expected to be difficult and lead to an increase in the number of insertion attempts.

Ultrasound (US) guidance allows CVC procedure more safely than conventional anatomic landmark techniques.^[1]^ Advancements in US imaging devices and access techniques have reduced mechanical complications encountered in clinical practice. Therefore, US-guided CVC insertion is highly recommended in practical guidelines when the internal jugular vein (IJV) is selected for cannulation.^[2]^ However, even if CVCs are inserted using real-time US guidance, which is the most reliable method, CVC malposition or vascular penetration cannot be prevented completely.

We report our experience with 2 cases of unexpected mechanical complications during CVC insertion despite the use of real-time US. These cases demonstrate that posterior wall penetration during CVC insertion can be easily overlooked even when using US.

## 2 Case report

The patients gave consent for this report and the institutional review board of Kyungpook National University hospital approved this study.

### 2.1 Case 1

A 19-year-old woman (weight = 58 kg, height = 155 cm, body mass index = 24.1 kg/m^2^) with ependymoma was scheduled for elective craniotomy. She presented no history of surgery or comorbidities. No abnormalities were detected on preoperative evaluation or physical examination. General anesthesia was maintained with total intravenous anesthesia with propofol and remifentanil. CVC cannulation in the right IJV was planned after endotracheal intubation.

**Figure 1:**
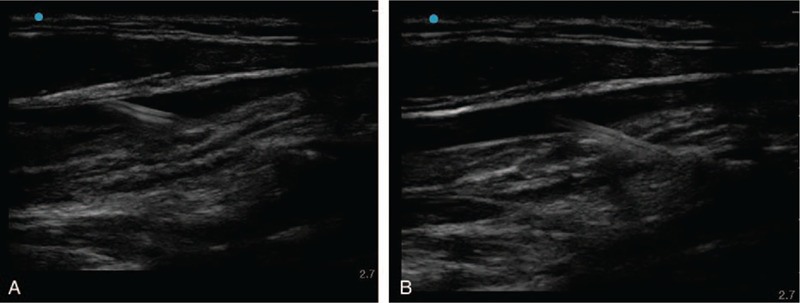
The guidewire penetrated the posterior wall of the right internal jugular vein.

**Figure 2:**
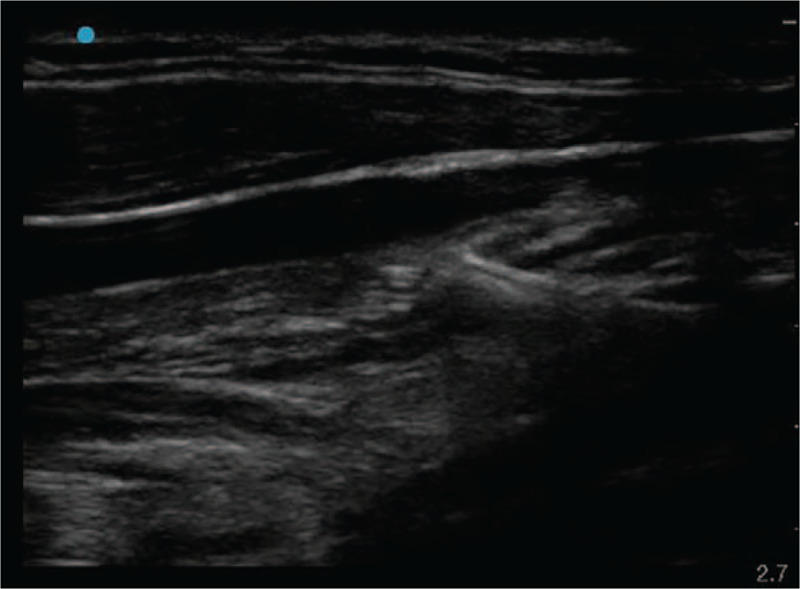
Broken J tip of the guidewire was embedded in the posterior wall of the right internal jugular vein. Coil filament outside of the core wire was still connected.

The patient's head was turned to the left, and the Trendelenberg position was assumed. The operator identified the US short-axis view to examine the location, diameter, and caliber of IJV. Simultaneously, patency and compressibility were checked. IJV was then punctured on the first attempt using out-of-plane real-time US guidance. After confirming blood aspiration, the guidewire was inserted using the Seldinger technique. When the guidewire was inserted about 10 cm, transient resistance was felt, and the insertion was stopped immediately. We attempted to withdraw the guidewire through the needle, but it was stuck in the vascular wall on the first attempt (Fig. 1). After a second attempt, the operator felt a sudden breakage in the guidewire, and it was pulled about 3 cm. An assistant examined the puncture site using real-time US while the operator maintained the state of the needle and guidewire. US revealed that the tip of the guidewire was located in the soft tissue through the blood vessel (Fig. 2).

**Figure 3:**
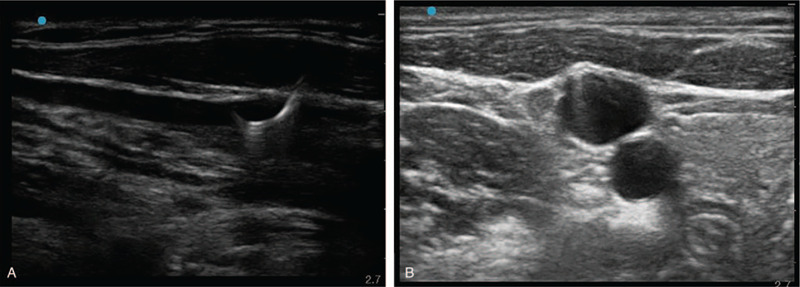
Broken J tip of the guidewire was withdrawn using real-time ultrasound guidance. Coil filament outside of the core wire was still connected.

**Figure 4:**
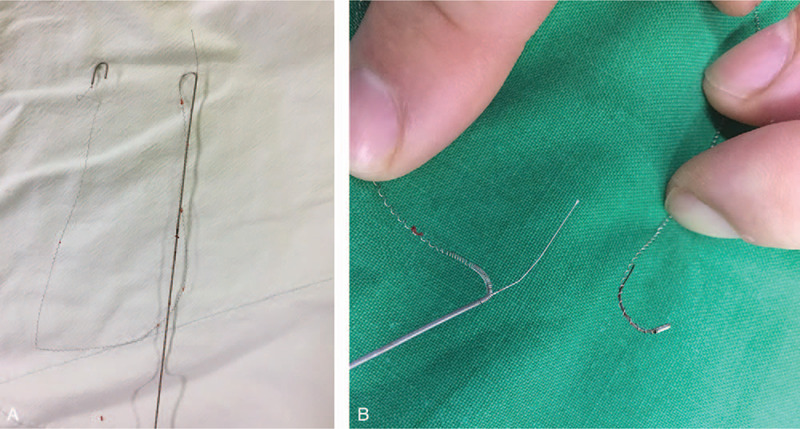
The guidewire was completely removed, and the outer coil filament was intact.

We immediately consulted a vascular surgeon. The surgeon noticed that the internal core of the guidewire was split into 2 segments. The external spring was still connected and unwound gradually when we pulled it. We planned to proceed with emergency surgery if the guidewire was broken but first attempted manual extraction. We also used real-time US to monitor continuity. The assistant displayed the US image in the long-axis view, and the vascular surgeon grasped the needle and guidewire simultaneously and slowly pulled it out (Fig. 3). The guidewire was completely removed (Fig. 4), and there was no hematoma or vascular injury after sufficient compression. After hemostasis, CVC was inserted into the contralateral IJV using real-time US. Surgery was completed without any specific events. Outpatient follow-up after discharge did not show any complications.

### 2.2 Case 2

A 50-year-old woman (weight = 51.6 kg, height = 155.7 cm, body mass index = 21.3 kg/m^2^) with ovary cancer was scheduled for elective debulking surgery. The patient presented no medical history. No abnormalities were noted on physical examination. After induction of general anesthesia, CVC insertion was planned to prepare for massive bleeding. The patient was placed in the Trendelenberg position. US short-axis images were used to assess the patency of the right IJV and surrounding structures before needle insertion. The needle was advanced in the short-axis view of real-time US using the one-handed technique. After confirming that the blood was aspirated, the guidewire was inserted using the Seldinger maneuver without US guidance. US was used to monitor the location of the guidewire in the IJV lumen, and a 3-lumen CVC was inserted up to 15 cm without any problems. All 3 lumens showed blood aspiration before connecting the fluid lines. The proximal lumen was connected to the pressure kit transducer for central venous pressure (CVP) monitoring, and CVP waveform appeared to be slightly overdamping. After zeroing, the CVP value was within 10 to 15 mmHg. However, a typical waveform was still not detected. Other intravenous fluids connecting to CVC were infused properly.

The operation lasted over 2 hours, and self-respiration continued to recover excessively, even though 0.5 mg/kg neuromuscular blocking agent was infused through the CVC line. Because there were no abnormal findings on the anesthetic machine or other monitors, CVC malposition was suspected and we checked CVC lumens. Clear fluid was aspirated through the distal lumen of CVC instead of blood, and the other 2 lumens were not aspirated.

**Figure 5:**
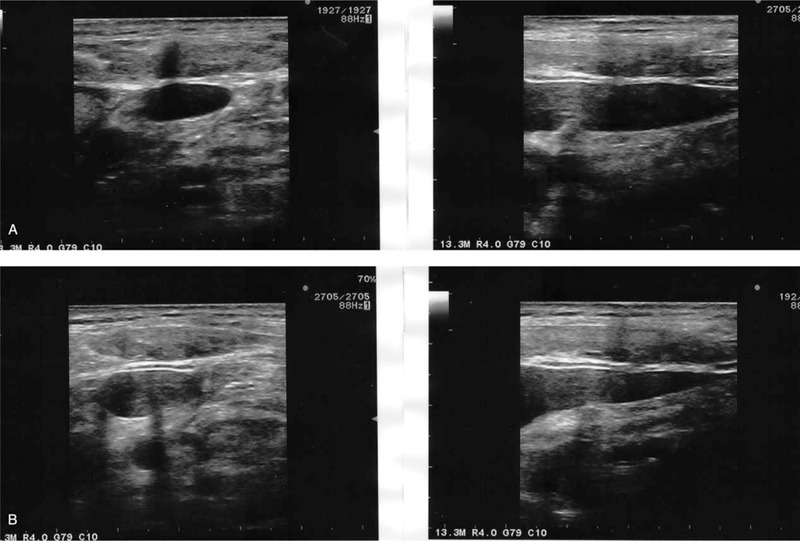
Central venous catheter was misplaced through the posterior wall of the internal jugular vein.

**Figure 6:**
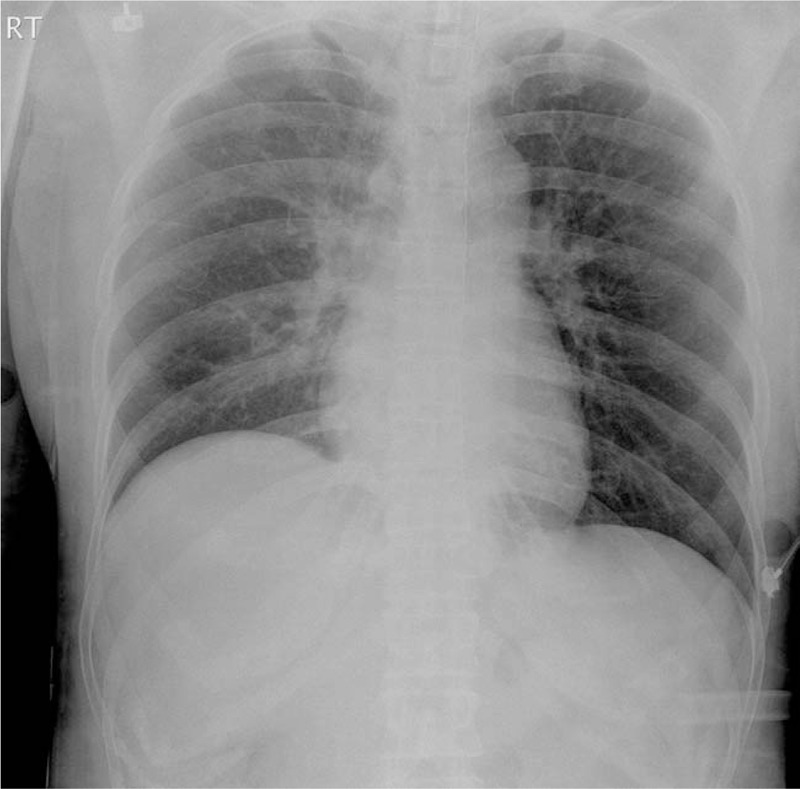
Chest radiography after removing the central venous catheter.

US revealed malpositioned CVC penetrating IJV (Fig. 5). We could not detect CVC tip. A thoracic surgeon suspected that CVC was entering the interpleural space. All fluid volumes injected through CVC were almost aspirated, and CVC was then removed under the attendance of a thoracic surgeon. Chest radiography performed immediately after hemostasis showed haziness in the entire right lung (Fig. 6). Consistently, arterial blood gas analysis showed normal results. After accessing the additional peripheral 18 G intravenous line, the operation was completed after nearly 6 hours. The patient was extubated without any difficulty and transferred to the ward after sufficient observation in the postanesthetic care unit. The patient was discharged 1 week later without any complications.

## 3 Discussion

Both patients in the present report showed a normal body mass index and preprocedural US findings. We expected no difficulties with CVC insertion, and the right IJV was punctured on the first attempt using real-time US. The operators were anesthesiology residents with ≥1 year of US experience. In the first case, the operator felt the resistance when the guidewire was inserted, and US confirmed that it was embedded instantly in the IJV posterior wall. Unfortunately, in the second case, the operator did not detect any resistance from guidewire and CVC insertion. After introducing the guidewire, the operator scanned the puncture site. In both long- and short-axes views, the guidewire appeared to be in the IJV lumen, although CVC had been malpositioned.

The availability of US has increased in many hospital departments, and US imaging devices can easily and accurately provide visualization of the great vessels and surrounding structures, even by novice users. US guidance has been included in standard protocols. There are several methods of US guidance, such as Doppler, real-time, and static imaging. During CVC insertion, a linear array probe is usually preferred, and this probe can scan to a depth of 20 to 50 mm.^[3]^ US is associated with fewer puncture attempts, lower technical failure rate, fewer mechanical complications, and shorter access time.^[1,4]^ However, for those unfamiliar with US guidance, the operator can be easily distracted by the direction of needle in the other hand and the two-dimensional images, particularly when using the one-handed method. As a result, the tip of the needle may not be properly recognized, or the direction of the needle may not be controlled as intended, causing the needle tip to leave the imaging plane and penetrate the vein or puncture the artery.

Although there have been many reports on mechanical complications during CVC insertion without US, adverse events related to the penetration of the posterior wall using real-time US guidance are underreported. In the out-of-plane approach of real-time US guidance, the incidence of posterior IJV wall penetration was 21%.^[5]^ This result illustrated that posterior IJV wall was still punctured despite real-time US guidance. As shown in our patients, puncture of the posterior IJV wall may be exacerbated by a guidewire lodged through the vessel wall or a CVC penetrating into the thorax. Moreover, in the classical head-rotated position, >90% of IJV is located in the anterior portion of the carotid artery, which means that accidental puncture of the carotid artery is possible even with US guidance.^[6]^

Methods to decrease the incidence of posterior IJV wall puncture using real-time US guidance are available. First, an in-plane approach, in which the entire needle shaft can be visualized and the needle tip never unintentionally penetrates the posterior vessel wall, is hypothetically advantageous. We used the out-of-plane approach in both cases reported here. This approach can puncture the vein more easily, and it is the most common approach for novice users.^[7]^ However, the out-of-plane approach is associated with a high risk of accidental penetration of the posterior IJV wall because only a cross-section of the needle is seen in plane.^[8]^ Using the out-of-plane approach, penetration of the posterior wall often occurs, particularly in patients with a small IJV diameter or easily collapsed IJV with hypovolemic status.^[9]^ The in-plane approach needs more practice than the out-of-plane approach to align the US beam and needle. The trajectory of the needle or catheter can also be disoriented in the long-axis view. Allowing the operator to become completely familiar with the in-plane technique through simulation training before applying it to a patient can reduce technical errors and complication rates.^[10]^ Second, the bevel-down approach may decrease the rate of IJV penetration compared with the conventional bevel-up approach. The sharp point of the needle tip only faces the posterior wall during IJV collapse, which leaves the posterior wall intact.^[11]^ This method has been used for arterial line or pediatric intravenous line puncture to reduce posterior wall penetration, because of its small diameter.^[12,13]^ Using all these methods together, even if the needle tip is located in the lumen of IJV and blood aspiration is confirmed on US, operators should keep track of the location of the needle tip and guidewire in the vessel lumen during the entire procedure.

Our cases also indicate that even after CVC insertion, US does not guarantee maximum protection if CVC is kinked, lodged, or malpositioned. To ensure appropriate catheter placement even with US guidance, several supportive measures have been suggested. First, although there is no doubt that real-time US-guided insertion has been successfully applied, we must monitor CVP waveform.^[14]^ In addition to CVP waveform, poor blood aspiration after monitoring the line connection and an abnormal CVP value may indicate CVC malposition. Second, a chest radiograph should be obtained as soon as possible after catheterization. It is better to confirm the position of the guidewire before insertion of a dilator or large-bore catheter over a wire. This will not be possible in most anesthetic conditions; therefore, chest radiography is recommended as soon as possible after surgery.^[2]^ Third, transthoracic or transesophageal echocardiography can also be used. Given the limitations of CVC insertion under real-time US guidance, echocardiography can be used to compensate for the conventional method. Transthoracic or transesophageal echocardiography can also be used to confirm the location of the guidewire or CVC tip. However, transthoracic or transesophageal echocardiography requires a trained assistant to properly scan the images, and transesophageal echocardiography can be used only in patients with an esophageal probe.^[15,16]^ Other methods that can be used as an indicator of proper catheterization include venous blood gas analysis, fluoroscopy, and continuous electrocardiography. Before using CVC, we must confirm the accurate CVC position using various methods.

## 4 Conclusion

In summary, we experienced posterior IJV wall penetration during CVC insertion using the out-of-plane approach of real-time US, which resulted in significant adverse events including guidewire breakage and CVC malposition. Real-time US guidance for vascular access has been used in clinical practice, and the benefits of US should be emphasized. At the same time, it is important to recognize that the risk of posterior IJV wall penetration is not eliminated with US guidance. During CVC insertion with real-time US, the in-plane and bevel-down approaches can be advantageous to prevent the penetration of the posterior IJV wall, and the position of CVC must be confirmed via supplementary monitoring, such as CVP waveform, chest radiography, and echocardiography.

## Author contributions

**Conceptualization:** Kyung-Hwa Kwak.

**Data curation:** Jeong Eun Lee.

**Formal analysis:** Jeong Eun Lee.

**Methodology:** Kyung-Hwa Kwak.

**Resources:** Myeong Jin Kim.

**Supervision:** Kyung-Hwa Kwak.

**Visualization:** Jeong Eun Lee.

**Writing – original draft:** Jeong Eun Lee.

**Writing – review & editing:** Kyung-Hwa Kwak.
